# Editorial: Designing Technologies for Youth Mental Health

**DOI:** 10.3389/fpubh.2020.00045

**Published:** 2020-02-25

**Authors:** Nilufar Baghaei, John A. Naslund, Sylvia Hach, Hai-Ning Liang

**Affiliations:** ^1^Department of Information Technology, Otago Polytechnic Auckland Campus, Auckland, New Zealand; ^2^School of Natural and Computational Sciences, Massey University, Auckland, New Zealand; ^3^Global Health and Social Medicine, Harvard Medical School, Boston, MA, United States; ^4^Clinical Research, Unitec Institute of Technology, Auckland, New Zealand; ^5^Department of Computer Science and Software Engineering, Xi'an Jiaotong-Liverpool University, Suzhou, China

**Keywords:** editorial, mental health, technology innovation, design, user experience (UX)

Mental health conditions pose a major challenge to healthcare providers and society at large (1, 2). The World Health Organization predicts that by the year 2030, mental health conditions will be the leading burden of disease globally (2). Mental health services across all countries are struggling to meet the needs of users and arguably fail to reach the majority of those in need. Preventative and early intervention, support and education can have significant positive impact on a person's prognosis, particularly important in affecting outcomes for young people (3). Co-designed solutions to improve resilience and well-being in young people have specifically been recognized as part of the NZ National Suicide Prevention Strategy (4).

Over the last few years there has been a growing interest in technologies for educating and supporting mental health, both as a maintenance strategy and intervention tool. Advances in technology have created opportunities for collaboration between Technology and Health researchers, and practitioners to design and develop tools to train and support healthcare providers, connect users with healthcare providers, provide access to affordable self-assessment and to provide treatment or preventative strategies. The use of technology can provide a greater degree of anonymity than what has been possible with existing health systems, which mostly focus on dealing with diagnosable cases. Technology is often considered an advantage for offering new opportunities in reaching individuals who might not otherwise seek help due to fear of being stigmatized. The use of technology in mental health has recently attracted a lot of attention by researchers and clinicians. It can potentially create new effective care models in the wider context of positive lifestyle changes, and prevention of, education about and support for individuals affected by mental health conditions especially sleep disorders, eating disorders, mood disorders, addictive behaviors, and substance abuse disorders.

In this special issue Designing Technologies for Youth Mental Health (https://www.frontiersin.org/research-topics/8765/designing-technologies-for-youth-mental-health) in *Frontiers in Public Health*, we bring together a collection of five interesting research studies from diverse regions, including New Zealand (5), the United States (6), Japan (7, 8), and India (9) that are focused on the role of emerging digital technologies for advancing mental health promotion and treatment efforts for youth. Importantly, we can place the studies included in this special issue along a continuum to illustrate the progression from initially establishing the viability of digital technology as a potential strategy for targeting mental health concerns by eliciting the preferences of young people regarding usability, acceptability, and specific design features of these digital platforms. This work then leads to conducting formal pilot testing of prototypes of digital mental health interventions.

The first study focused on studying game interface preferences for users with a history of mental health. Baghaei et al. (5) presented MoodJumper, a prototype Android mobile game, which enables players to jump to the top of the level by steering the avatar from platform to platform, gradually gaining height and collecting coins on the way up. They conducted a preliminary study, during which participants were able to modify different settings of the game (background color, dark/light, character movement, gender, and music), while their gaming behavior was tracked. The results showed that, regardless of self-reported history of mood disorder, majority of participants prefer the dark and colored layout setting and there were no differences in gaming variables including session duration and high scores. This represents a first indication that history of mood disorder does not affect user preferences for game interface settings. It will be important to follow up this study with data on users currently affected by low mood.

Aschbrenner et al. (6) employed a combination of surveys and focus group interviews with adolescents receiving public mental health services to better understand their views and interests related to technology use for supporting mental health care. This mixed methods study conducted in public mental health settings in the Northeastern United States found near ubiquitous use of smartphone apps and social media among adolescent participants, and that participants expressed preferences for mobile health coaching support and structured online peer-to-peer support for mental health promotion interventions.

In the first of two studies using android robots from Japan, Yoshikawa et al. (7) further our understanding of the role of emerging technologies for facilitating communication among adolescents with autism spectrum disorders (ASDs) through the observation of participants' patterns of eye contact and interactions with an android robot. This study found similarities in how participants with and without ASD made eye contact with an android robot. They reported that individuals with ASD appeared to look at the eyes of a human less frequently when compared to individuals without ASD. In the second study from Japan, Kumazaki et al. (8) employed role-play activities with an android robot to help adolescents with ASDs better recognize gestures and other interpersonal characteristics of communication in the context of important life skills such as interviewing for a job. Interestingly, in this study individuals with ASD worked in pairs, taking turns acting as the interviewee or operating the android robot that acted as the interviewer (8). By creating this unique role play training environment, participants appeared to better understand the interviewer and expressed greater self-confidence. During the 1-year follow up period after completion of the role play sessions, five out of eight participants completed job interviews and successfully obtained employment demonstrating that use of android robot technology in a controlled setting can potentially translate to real world behavior changes and acquisition of important life skills for participants with ASDs (8).

The fifth study focused on advancing our understanding of the role of digital technologies for mental health promotion in public school settings in a low-resource setting in India. Gonsalves et al. (9) used multiple iterative steps including focus group discussions with adolescents and service providers, co-design workshops, and user testing of early prototypes toward designing a blended game-based problem-solving intervention for adolescent mental health. By employing a collaborative person-centered approach, it was possible for the researchers to optimize various features throughout the development of this digital game-based mental health intervention. This included simplification of language to meet the needs of a lower literacy student population, accommodating the need for offline access to the digital intervention content given the poor Internet connectivity in the region, incorporating sufficient risk assessment and management features into the mobile app to respond to participants' low mood, and the inclusion of low-intensity human support to offer instruction and personalized support to participants as they complete the game (9).

These studies demonstrate that technologies not only appear feasible for youth mental health, but may also be a preferred medium for accessing support among many. Interestingly, across the different studies and contexts, several adolescent participants emphasized an interest in maintaining human connection, either through access to peers, coaches, or other forms of human support, suggesting that a purely digital format for youth mental health interventions may not be ideal for all young people. These initial studies emphasize the importance of carefully conducted pilot and exploratory studies to establish feasibility and acceptability, and in particular, the value of closely involving young people and other relevant stakeholders throughout the design, development and initial testing of interventions for youth mental health (10–12). As the field of digital technologies for youth mental health is still in the early stages, continued efforts are necessary to consider persistent inequalities in access to digital devices due to poverty and other social inequities, and to strive toward inclusiveness in the design and development of digital interventions (13). Such efforts hold potential to promote adoption across diverse contexts, regions, and cultures, and ensure generalizability and sustainability of digital mental health interventions toward reducing the burden of mental disorders globally.

## Author Contributions

All authors listed have made a substantial, direct and intellectual contribution to the work, and approved it for publication.

### Conflict of Interest

The authors declare that the research was conducted in the absence of any commercial or financial relationships that could be construed as a potential conflict of interest.
